# Functional Characterization of MODY2 Mutations Highlights the Importance of the Fine-Tuning of Glucokinase and Its Role in Glucose Sensing

**DOI:** 10.1371/journal.pone.0030518

**Published:** 2012-01-24

**Authors:** Carmen-María García-Herrero, Oscar Rubio-Cabezas, Sharona Azriel, Angel Gutierrez-Nogués, Angel Aragonés, Olivier Vincent, Angel Campos-Barros, Jesús Argente, María-Angeles Navas

**Affiliations:** 1 Departamento de Bioquímica y Biología Molecular III, Facultad de Medicina, Universidad Complutense de Madrid and Instituto de Investigación Sanitaria del Hospital Clínico San Carlos, Madrid, Spain; 2 Centro de Investigación Biomédica en Red de Diabetes y Enfermedades Metabólicas (CIBERDEM), www.ciberdem.net, Instituto de Salud Carlos III, Madrid, Spain; 3 Servicio de Endocrinología, Hospital Infantil Universitario Niño Jesús, Instituto de Investigación La Princesa and Departamento de Pediatría, Universidad Autónoma de Madrid, Madrid, Spain; 4 Centro de Investigación Biomédica en Red de la Fisiopatología de la Obesidad y Nutrición (CIBEROBN), www.ciberobn.es, Instituto de Salud Carlos III, Madrid, Spain; 5 Servicio de Endocrinología, Hospital Universitario Infanta Sofía, San Sebastián de los Reyes, Madrid, Spain; 6 Servicio de Pediatría, Hospital Virgen de la Salud, Toledo, Spain; 7 Instituto de Investigaciones Biomédicas Alberto Sols, Consejo Superior de Investigaciones Científicas-Universidad Autónoma de Madrid, Madrid, Spain; Institute of Enzymology of the Hungarian Academy of Science, Hungary

## Abstract

Glucokinase (GK) acts as a glucose sensor in the pancreatic beta-cell and regulates insulin secretion. Heterozygous mutations in the human GK-encoding *GCK* gene that reduce the activity index increase the glucose-stimulated insulin secretion threshold and cause familial, mild fasting hyperglycaemia, also known as Maturity Onset Diabetes of the Young type 2 (MODY2). Here we describe the biochemical characterization of five missense GK mutations: p.Ile130Thr, p.Asp205His, p.Gly223Ser, p.His416Arg and p.Ala449Thr. The enzymatic analysis of the corresponding bacterially expressed GST-GK mutant proteins show that all of them impair the kinetic characteristics of the enzyme. In keeping with their position within the protein, mutations p.Ile130Thr, p.Asp205His, p.Gly223Ser, and p.His416Arg strongly decrease the activity index of GK, affecting to one or more kinetic parameters. In contrast, the p.Ala449Thr mutation, which is located in the allosteric activator site, does not affect significantly the activity index of GK, but dramatically modifies the main kinetic parameters responsible for the function of this enzyme as a glucose sensor. The reduced Kcat of the mutant (3.21±0.28 s^−1^ vs 47.86±2.78 s^−1^) is balanced by an increased glucose affinity (S_0.5_ = 1.33±0.08 mM vs 7.86±0.09 mM) and loss of cooperativity for this substrate. We further studied the mechanism by which this mutation impaired GK kinetics by measuring the differential effects of several competitive inhibitors and one allosteric activator on the mutant protein. Our results suggest that this mutation alters the equilibrium between the conformational states of glucokinase and highlights the importance of the fine-tuning of GK and its role in glucose sensing.

## Introduction

Glucokinase (GK) plays a key role as the pancreatic beta-cell glucose sensor by integrating blood glucose levels and glucose metabolism with insulin secretion [1]. Besides the beta-cell, GK is expressed in other neuroendocrine cells and in hepatocytes, which participate in a network of cells involved in the body glucose homeostasis [2]. This enzyme (also called hexokinase IV) is one of the four hexokinase isoforms that catalyze the first reaction of glycolysis converting glucose into glucose-6-phosphate (G6P) with ATP as second substrate. The specific function of GK as a glucose sensor is based on the particular regulatory characteristics of this enzyme, as compared with the other hexokinase isoforms, which are low affinity for glucose (S_0.5_ 7–9 mM, cooperativity with this substrate (Hill coefficient≈1.7) and lack of end-product inhibition at physiological concentrations of G6P. In addition, and also in contrast to other hexokinase isoforms, glucokinase activity might be modified by protein interaction partners that can modulate the catalytic activity and the intracellular distribution of the enzyme in beta-cells and/or hepatocytes (for review see [3] [4] [5]. Yet, the best known regulator of GK in the liver is the glucokinase regulatory protein (GKRP) that acts as a competitive inhibitor with respect to glucose and regulates the nucleo-cytoplasmic localization of the enzyme to stabilize a protein reservoir of the enzyme in the nucleus when its activity is not necessary [6] [7] [8].

The crucial role of GK on beta-cell function is illustrated by the fact that over 600 mutations in the *GCK* gene cause different monogenic glycaemic disorders (for review see [9]). Thus, heterozygous activating mutations cause persistent hyperinsulinemic hypoglycaemia of infancy (HI). In contrast, homozygous inactivating mutations cause complete GK deficiency and permanent neonatal diabetes mellitus (PNDM), whereas heterozygous inactivating mutations cause familial, mild fasting hyperglycaemia also known as maturity-onset diabetes of the young type 2 (MODY2; [10] [11]). MODY2 patients show mild fasting hyperglycaemia (5.5–8.0 mM) present from birth but usually are asymptomatic and most remain undiagnosed until later in life [12]. These patients show little deterioration with age and usually do not require any specific treatment [13]. The principal pathophysiological mechanism of altered glycaemia in patients with GK mutations is a beta-cell dysfunction characterized by a modification in the blood glucose threshold that triggers insulin secretion, which is consistent with a defect in glucose sensing [14] [15]. In addition, abnormalities in liver glucose metabolism contribute to the hyperglycaemia in patients with MODY2 [16]. Due to its central role in controlling body glucose homeostasis, GK has been considered as an outstanding drug target for developing new antidiabetic therapies and several small molecular activators (GKAs) have been discovered (for review see [5] [17]).

The determination of the human GK crystal structure defined the active and allosteric activator sites and revealed the basis for the catalytic mechanism of this enzyme thus allowing researchers to analyze disease-associated mutations at the molecular level [18]. The functional characterization of naturally occurring GK mutations has contributed to better knowledge of the biochemistry of this enzyme. Activating mutations involved in HI increase glucose affinity and most of them are clustered at the allosteric activator site where GKAs bind [9] [18]. In contrast, hyperglycaemia causing mutations are located throughout the protein and most of them show a lower activity index, due to a reduction in substrate affinity combined or not with decreased catalytic turnover, which leads to an increased threshold for glucose-stimulated insulin release [9] [19].

The present work reports the functional analysis of five missense GK mutations: p.Ile130Thr, p.Asp205His, p.Gly223Ser, p.His416Arg and p.Ala449Thr. The biochemical characterization of these mutations shows that most of them are inactivating mutations decreasing the activity index of the enzyme. Unexpectedly, the p.Ala449Thr mutation shows an overall activity index that does not differ significantly from that of the wild type. In contrast, it dramatically modifies the main kinetic parameters responsible for the function of GK as a glucose sensor. By studying the effect of one allosteric activator and some competitive inhibitors on enzymatic activity, we analyzed the mechanisms by which this mutation, located in the allosteric activator site, impairs GK fine tuning.

## Materials and Methods

### Patients and mutation analysis of the GCK gene

The diabetic patients involved in this study were referred to our laboratories for molecular testing of MODY2. The clinical diagnosis was made using classical criteria [11]. All families were of Spanish Caucasian descent with the exception of patient 4 who was of Asian descent. Written informed consent was obtained from the subjects or their parents. The studies were performed according to the Declaration of Helsinki as revised in 2000 and approved by the local Clinical Research Ethical Committees of the two centres which conducted human genetic studies (Hospital Clinico San Carlos/Universidad Complutense de Madrid and Hospital Infantil Universitario Niño Jesús, Madrid).

Mutation analysis of the *GCK* gene was performed as previously described [20] [21].

### Production of recombinant wild-type and mutant glutathionyl-S-transferase-glucokinase (GST-GK) fusion proteins

Recombinant human wild-type beta-cell glucokinase fused to GST (GST-GK) was prepared as described previously [22]. MODY-associated mutations were introduced into the GST-GK construct by PCR using the QuikChange® II Site Directed Mutagenesis Kit (Stratagene, La Jolla, Calif., USA). To generate missense mutations p.Ile130Thr, p.Asp205His, p.Gly223Ser, p.His416Arg and p.Ala449Thr the following oligonucleotides were used, respectively: 5′-ctacatctctgagtgcacctccgacttcctggac-3′, 5′-gatgtggtggccatggtgaatcacacggtggccacg-3′, 5′-gaggtcagcatgatcgtgggcacgggctgcaatgcatgctac-3′, 5′-ctccgtgtacaagctgcgcccaagcttcaaggag-3′, and 5′-gaggagggcagcggccggggcacggccctgctg-3′. Constructs carrying mutations p.Ile130Thr, p.Asp205His, p.Gly223Ser, p.His416Arg and p.Ala449Thr were checked by sequencing and digestion with ApaLI, NcoI, NsiI, HindIII and Eco52I, respectively. The expression and purification of fusion proteins from *E. coli* was performed as described previously [22]. Fusion proteins were stored at a concentration of about 1 mg/ml at −80°C in 30% glycerol, 50 mM glucose, 10 mM glutathione, 5 mM DTT, 200 mM KCl and 50 mM Tris/HCl pH = 8.0. Protein concentrations were determined using the Bio-Rad Protein Assay (Bio-Rad Laboratories, GmbH), with bovine serum albumin as standard. GST-GK purifications resulted in single bands of 75 kDa on Coomassie-stained SDS-PAGE.

### Kinetic and structural analysis of the GK protein

Glucokinase activity was measured spectrophotometrically on a spectrophotometer Uviconxl (Secoman), using an NADP^+^-coupled assay with glucose-6-phosphate dehydrogenase, as described previously [22]. The final reaction mixture contained 100 mM Tris pH 7.4, 150 mM KCl, 1 mM MgCl_2_, 1.4 mM ß-mercaptoethanol, 0.1% BSA, 5 mM ATP-MgCl_2_, 1 mM NADP^+^, and 1 U/ml glucose-6-phosphate dehydrogenase. Determination of kinetic parameters and thermal stability tests were performed as in [20]. Km for ATP was measured at a glucose concentration corresponding to the S_0.5_ value for GST-GK wild-type and mutant proteins (8, 9, 40, 70 and 3 mM, for wild-type, GST-GK(p.Ile130Thr), GST-GK(p.Gly223His), GST-GK(p.His416Arg) and GST-GK(p.Ala449Thr), respectively. The relative activity index was calculated as previously [19]. Concentrations of inhibitors causing 50% inhibition (I_50_) were derived from Dixon plots [23], using different concentrations of glucose (2, 5 and 8 mM for the wild-type protein, 12.5, 25 and 50 mM or 1, 1.5 and 2 mM for proteins carrying mutations p.Gly223Ser and p.Ala449Thr, respectively), and five concentrations of inhibitors (0.05, 0.1, 0.25, 0.5 and 1 mM for N-acetylglucosamine (NAG); 0.5, 1, 2, 5 and 10 mM for mannoheptulose (MH) and 1, 2, 5, 10 and 15 µM for palmitoyl-CoA). Inhibition by NAG and MH was determined under standard assay conditions with 50 nM glucokinase [22]. The inhibition by palmitoylCoA was determined in reaction mixtures containing 25 mM Hepes pH 7.1; 25 mM KCl; 1 mM NADP, 1 mM ATP-MgCl_2_; 2.5 mM MgCl_2_; 5 mM ß-mercaptoethanol, 1 U/ml glucose-6-phosphate dehydrogenase and 61 nM glucokinase [24]. Due to the low Kcat value of mutant GST-GK(pAla449Thr), the amount of protein used in inhibition assays was 180 nM to get sufficient activity to allow for accurate determination of the inhibitor effect. In these conditions, GK activity (U/mg of GST-GK(pAla449Thr)) in the absence of inhibitors was 1.3±0.08, 1.1±0.06 and 0.9±0.05 at 2, 1.5 and 1 mM of glucose, respectively for NAG and MH assays and 0.5±0.05, 0.33±0.03 and 0.27±0.03 at 2, 1.5 and 1 mM glucose, respectively for palmitoyl-CoA assay.

Purified human GKRP was kindly provided by KJ Brocklehurst (Cardiovascular and Gastrointestinal Department, AstraZeneca, Macclesfield, Cheshire SK10 4TG, UK). The effect of GKRP on glucokinase activity was determined as described [25]. The final reaction mixture contained 25 mM Hepes pH 7.1; 25 mM KCl; 1 mM NADP, 1 mM ATP-MgCl2; 2.5 mM MgCl2; 5 mM ß-mercaptoethanol, 1 U/ml glucose-6-phosphate dehydrogenase, 61 nM of GST-GK or 180 nM GST-GK(pAla449Thr), 5 mM glucose, GKRP (0 to 200 nM) and, when indicated, 10 µM sorbitol-6-phosphate (S6P) or 0.2 mM fructose-1-phosphate (F1P). In these conditions, GST-GK(pAla449Thr) activity in the absence of GKRP was 0.8±0.09 U/mg of protein.

One unit of GK is defined as the amount of enzyme that phosphorylates 1 µmol of glucose per min at 30°C.

GK activator LY2121260 was kindly provided by Elli Lilly and Co, Indianapolis, IN, USA. A stock solution of 1 mM LY2121260 was prepared in 12.5 mM Hepes pH 7.4 with 80% DMSO. Results are shown as means ± SEM, and statistical significance was analysed by the two-tailed Student's t-test. *P* values of <0.05 were considered statistically significant. The structural analysis of mutations was carried out using the crystal structure of human GK [18] and visualized using the Pymol Molecular Graphics System (Schrödinger).

### Two-hybrid analysis

Two-hybrid studies were performed in *S. cerevisiae* strain Y187 as described previously [20]. Plasmids encoding a GKRP fusion protein to the Gal4 Binding Domain (GBD) in the pGBKT7 vector (Clontech) and a fusion of GK to the Gal4 Activating Domain (GAD) in the pACTII (Clontech), have been described previously [22]. Plasmid encoding the GK(p.Ala449Thr) mutant derivative was derived from pACTII by inserting a BamHI-XhoI fragment from the GST-GK(p.Ala449Thr) construct containing the mutant GK coding sequence between the same sites of the polylinker.

## Results

### Clinical features of probands and mutations in the *GCK* gene

Table 1 shows the clinical presentation of probands in the 5 families, consistent with a clinical diagnosis of MODY2 in all cases. All of these patients were treated with diet alone. A *GCK* heterozygous missense mutation was identified in each family. Three mutations (p.Ile130Thr, p.Gly223Ser, and p.Ala449Thr) had been previously reported as a cause of MODY [9] [26]. Two mutations were novel (p.Asp205His, p.His416Arg). Proband 2 inherited the p.Asp205His mutation from his affected mother; it was also found in an affected sister but not in his unaffected father. Proband 4 had been adopted from China and DNA from her biological relatives was not available for testing.

**Table 1:** Clinical characteristics of probands and *GCK* mutations.

Family	Age at diagnosis/Current age (years)	Patient sex	BMI(kg/m^2^)	FPG(mM)	OGTT(mM)	HbA_1c_(%)	Nucleotide change	Protein change	Affected family members
1	3 yr3 mo/21	Female	15	6.1	5.8	5.8	c.389T>C	p.Ile130Thre	F, pGF
2	11 yr6 mo/19	Male	16.6	6.8	9.3	6.5	c.613G>C	**p.Asp205His**	M, S, mGF, mGM
3	13/19	Male	17	7.2	10.2	6.3	c.667G>A	p.Gly223Ser	F
4	1 yr11 mo/9	Female	17	6.3	11.1	5.7	c.1247A>G	**p.His416Arg**	Adopted
P99	27/31	Female	28	7.4	NA	NA	c.1345G>A	p.Ala449Thr	pGF, F

BMI: body mass index; FPG: fasting plasma glucose; OGTT: plasma glucose at 120 min after a standard oral glucose tolerance test (1.75 g per kg, máx. 75 g). Nucleotide numbering uses +1 as the A of the ATG initiation codon, based on the GenBank sequence # NM_000162. F, father; M, mother; S, sister; pGF, paternal grandfather; mGF, maternal grandfather; mGM, maternal grandmother. NA, not analysed. Underlined are those affected family members where mutation was checked by genotyping. Novel mutations are shown in bold.

### Biochemical analysis of recombinant mutant glucokinases

Recombinant wild-type GST-GK and mutants GST-GK(p.Ile130Thr), GST-GK(p.Asp205His), GST-GK(p.Gly223Ser), GST-GK(p.His416Arg) and GST-GK(p.Ala449Thr) were prepared in *E. coli*. Kinetic parameters of purified fusion proteins are shown in Table 2. Mutation p.Asp205His produced the strongest effect on GK, since this mutant enzyme showed no measurable activity despite the good protein yield obtained after purification. Mutants GST-GK(p.Ile130Thr), GST-GK(p.Gly223Ser) and GST-GK(p.His416Arg) showed an overall activity lower than 20, 10 and 0.5% of the wild-type protein respectively, which is reflected in the relative activity index (I_a_) values. Among these mutations p.Gly223Ser and p.His416Arg significantly affected all the kinetic parameters. Both mutations decreased the affinity of GK for both substrates glucose and ATP, and also decreased the catalytic constant (Kcat) and the Hill coefficient (nH) of the enzyme. In contrast, mutation p.Ile130Thr, which also decreased the Kcat and the nH, increased the affinity of GK for ATP without modifying the affinity for glucose.

**Table 2:** Kinetic constants of human recombinant wild type and MODY2 mutant beta-cell GST-GK fusion proteins.

Preparation	Protein yield (mg/l)	Kcat (s^−1^)	S_0.5_ for glucose (mM)	nH	Km for ATP (mM)	I_a_
Wild-type GST-GK(*n* = 7)	4.83±0.02	47.86±2.78	7.86±0.09	1.49±0.02	0.54±0.01	1.0±0.09
GST-GK (p.Ile130Thr)(*n* = 4)	14.3±0.14	2.88±0.29*	8.59±0.45	1.01±0.02*	0.13±0.02**	0.17±0.03*
GST-GK (p.Asp205His)(*n* = 2)	7.7/3.3	No activity at all				
GST-GK (p.Gly223Ser)(*n* = 5)	7.75±0.07	12.90±0.52*	44.0±3.81*	1.13±0.01*	1.60±0.1*	0.064±0.010*
GST-GK (p.His416Arg)(*n* = 4)	6.55±0.003	1.02±0.08*	67.9±2.27*	1.00±0.02*	3.74±0.10*	0.003±0.0004*
GST-GK (p.Ala449Thr)(*n* = 6)	9.65±0.065	3.21±0.28*	1.33±0.08*	0.95±0.02*	1.81±0.08*	1.23±0.16

Data represent means ± SEM of *n* separate experiments from at least two independent enzyme purifications. The Hill coefficient (nH) and the relative activity index (I_a_) are unit less. (*) *p*<0.0007; (**) *p* = 0.012.

**Figure 1:**
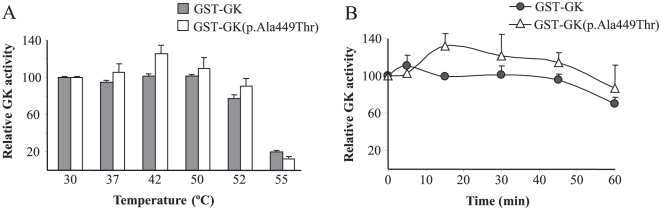
Effect of temperature on the stability of the GST-GK(p.Ala449Thr) protein. Stock enzyme solutions were diluted to 250 µg/ml in buffer containing 30% glycerol, 50 mM glucose, 10 mM glutathione, 5 mM dithiothreitol, 200 mM KCl and 50 mM Tris/HCl, pH 8.0. A) The enzyme solutions were incubated for 30 min at different temperatures ranging from 30 to 55°C and then assayed at 30°C as described in Material and Methods. B) The enzyme solutions were incubated for different periods of time from 5 to 60 min at 50°C. Means and SEM of three independent enzyme preparations are shown.

Mutant GST-GK(p.Ala449Thr) had an *in vitro* activity index slightly higher than the wild-type protein, although this difference was not statistically significant (*p* = 0.11). However, and despite this apparent normal activity level, this mutation affects all the kinetic parameters of GK individually. The mutant protein GST-GK(p.Ala449Thr) lacked the cooperativity for glucose, as shown by a significantly decreased Hill coefficient. The Kcat of this mutant was reduced to 7% of the wild-type value and the affinity for ATP was decreased more than 3-fold. In contrast, and unusually for MODY2 mutations, affinity for glucose was strongly increased in about 5-fold. The protein stability of mutant GST-GK(p.Ala449Thr) was tested at different temperatures and compared to that of the wild-type fusion protein. Figure 1A shows that the activity of both wild type and mutant GST-GK(p.Ala449Thr) was not significantly altered after 30 minutes of incubation up to 52°C but drastically fell at 55°C. Additionally, no significant differences in the relative activity were observed between both proteins when incubated up to 60 min at the constant temperature of 50°C (Figure 1B). These results indicate that mutation p.Ala449Thr did not confer thermal instability.

**Figure 2:**
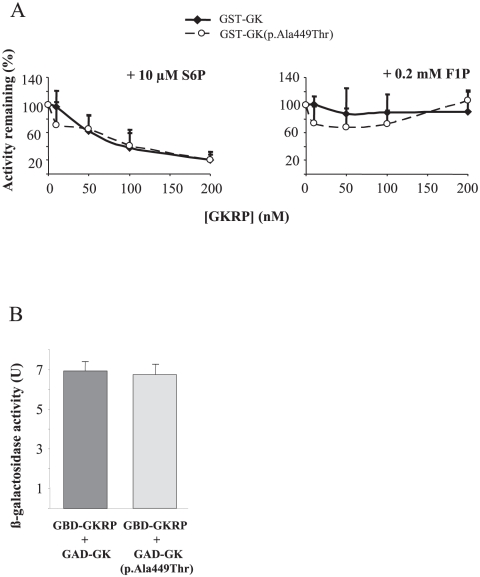
Effect of mutation p.Ala449Thr on the GK interaction with GKRP. A) Inhibition of glucokinase activity by human GKRP. Enzyme activity was measured at 5 mM glucose as described in Material and Methods, in the presence of 10 µM S6P (left panel) or 0.2 mM F1P (right panel). Results are means ± SEM for three independent enzyme purifications assayed in triplicate. B) Two-hybrid interaction of GBD-GKRP with GAD-GK or GAD-GK(p.Ala449Thr) mutant. Yeast strain Y187 was used, and fusion proteins were expressed from pGBKT7 and pACTII derivatives. Values are means ± SEM from ß-galactosidase activity of six independent transformants. In control experiments, GBD-GKRP did not interact with GAD and GAD-GK did not interact with GBD [22].

**Figure 3:**
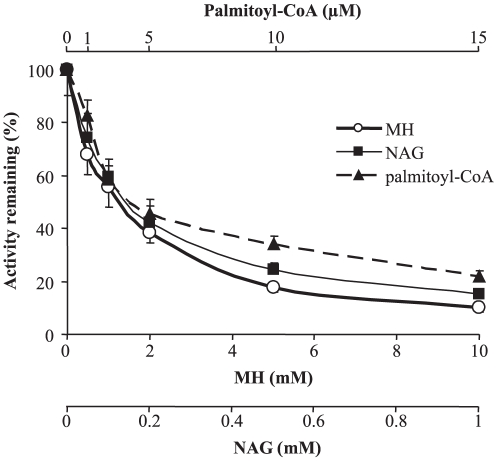
Inhibition of GK activity by mannoheptulose (MH), N-acetylglucosamine (NAG) and palmitoyl-CoA. The GST-GK enzyme was assayed at 5 mM glucose with the indicated concentration of inhibitors as described in Material and Methods. Means and SEM of three independent enzyme preparations are shown.

To further investigate the mechanism by which mutation p.Ala449Thr increased the affinity of GK for glucose, we tested the effects on both mutant and wild-type enzymes, of some competitive GK inhibitors such as N-acetylglucosamine (NAG), mannoheptulose (MH), palmitoyl-CoA, as well as the glucokinase regulatory protein (GKRP). Palmitoyl-CoA and GKRP bind to distinct allosteric sites, whereas glucose analogues NAG and MH bind to the catalytic site of the open and closed conformations, respectively [27]. It has been described that mutations of residues in the catalytic site affect the affinities for glucose, MH and NAG, whilst mutations of residues distant form the catalytic site affect the affinity for glucose and MH but not for NAG [28], [29], [27]. In addition, it has been previously proposed that differences in GKRP binding affinity between GK mutants reflect the mechanism by which mutations increase the glucose binding affinity [30]. As shown in Figure 2A, enzymatic inhibition by GKRP in the presence of either S6P or F1P was the same for the wild type and GST-GK(p.Ala449Thr) mutant. Lack of effect of this mutation on the GK-GKRP protein-protein interaction was corroborated by the two-hybrid system in yeast (Figure 2B). The sensitivity of GK to NAG, MH and palmitoyl-CoA was measured for both GST-GK(p.Ala449Thr) and GST-GK(p.Gly223Ser) mutant proteins, the latter showing a strongly decreased glucose affinity but still measurable enzymatic activity. The inhibition profiles for GST-GK activity are shown in Figure 3. Table 3 shows that the sensitivity of GK to these inhibitors, which is reflected by the I_50_ value, is significantly affected for the GK(p.Gly223Ser) mutant with respect to the wild-type enzyme. In contrast, mutation p.Ala449Thr did not affect the inhibition by NAG but decreased the I_50_ for MH and palmitoyl-CoA by about 35 and 26%, respectively.

**Table 3:** Effect of MODY2 mutations p.Gly223Ser and p.Ala449Thr on the affinity of GK for N-acetylglucosamine (NAG), mannoheptulose (MH) and palmitoyl-CoA.

Preparation	I_50_ for NAG (mM)	I_50_ for MH (mM)	I_50_ for palmitoyl-CoA (µM)
Wild-type GST-GK	0.21±0.04	1.15±0.37	3.06±0.27
GST-GK(pGly223Ser)	0.38±0.045**	3.55±0.63**	1.42±0.13**
GST-GK(p.Ala449Thr)	0.20±0.06	0.67±0.04**	2.24±0.14*

Concentrations of inhibitors causing 50% inhibition (I_50_) were derived from Dixon plots as described in Materials and Methods. Data represent means ± SEM of at least four separate experiments from at least two independent enzyme purifications. (**) *p*<0.006. (*) *p* = 0.03.

Finally, we studied the effects of the GK synthetic allosteric activator LY2121260 on the kinetic parameters of GST-GK(p.Ala449Thr) and compared them to those on the GST-GK wild-type protein (Table 4). For the wild-type fusion protein, the Kcat was increased by about 30% and the affinity for glucose was about 5 fold higher in the presence of 10 µM of this activator. In contrast, LY2121260 did not affect GST-GK(p.Ala449Thr) S_0.5_ but produced a slight recovery of Kcat up to about 20% of the normal wild-type value.

**Table 4:** Effect of GK synthetic activator LY2121260 on wild type and mutant GST-GK fusion proteins.

Preparation	Kcat (%)		S_0.5_ for glucose (mM)	
	Buffer	LY2121260	Buffer	LY2121260
Wild-type GST-GK	100±5.3	130.8±3.9**	7.90±0.3	1.7±0,1**
GST-GK(p.Ala449Thr)	13.9±0.8	19.4±1.5*	2.7±0.3	3.1±0.3

GK activity was measured in standard conditions, as described in Material and Methods, in the absence and presence of 10 µM GK activator. Since LY2121260 was dissolved in a buffer containing DMSO, all assays contained a final concentration of 0.8% DMSO. Data represent means ± SEM of at least four separate experiments from two independent enzyme purifications. Statistical significance has been estimated comparing values in the presence of LY2121260 versus their corresponding values in the presence of buffer. (**) *p*<0.005; (*) *p* = 0.023.

## Discussion

In this report we describe the functional analysis of five *GCK* mutations identified in probands with familial, mild fasting hyperglycaemia. The biochemical characterization of these mutations has shown that all of them impair normal glucokinase activity. The molecular analysis of these mutations revealed the particular defects caused in GK enzymatic activity.

**Figure 4:**
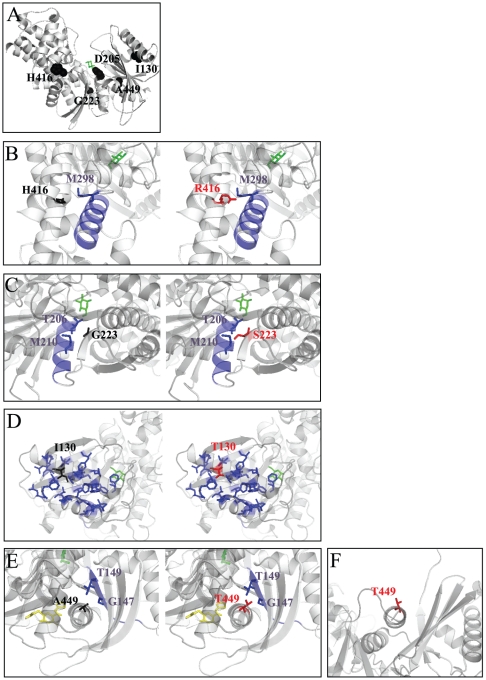
Localization of mutations in the structural model for beta-cell glucokinase. A) Localization of mutated residues in the closed conformation of GK. A to E) An enlargement of the region of interest is shown in each panel. F) Representation of mutation p.Ala449Thr in the super-open conformation of GK. Wild type residues are in black, whereas the mutated residues are magenta. Surrounding disturbing structures are in blue. Glucose is represented in green whereas activator compound A is in yellow. The closed (1V4S) and the super-open (1V4T) conformations of GK [18] are represented using the Pymol Molecular Graphics System (Schrödinger).

The structural data obtained from the free and substrate/activator bound GK crystals showed that this enzyme has a small and a large domain separated by a deep cleft where substrates bind [18]. Glucokinase may adopt three structural conformations. In the absence of glucose, GK is in an inactive *super-open* conformation. Following glucose binding, the enzyme undergoes a conformational change leading to, first, an intermediate *open* conformation and then, a fully active *closed* conformation, which binds ATP. The transition between the super-open and closed conformations involves the rotation and approximation of the small domain to the large one. The existence of GK in different conformations allows two catalytic cycles, fast and slow, that explain the sigmoidal response to glucose [18]. Asp205, His416 and Gly223 are highly conserved amino acids among glucokinases and hexokinases and, in agreement with their localization within or in close proximity to the catalytic site (Figure 4), are essential for GK activity as shown by the strongest overall activity defects produced by mutations p.Asp205His, p.His416Arg and p.Gly223Ser. Our results show that the novel mutation p.Asp205His abolishes GK enzymatic activity. This result was predictable since the interaction between Asp205 and glucose is necessary to trigger the conformational change between the inactive and active conformations of GK [18]. Mutations p.His416Arg and p.Gly223Ser severely inactivated the enzyme by impairment of all kinetic parameters. Gly223 and His416 do not interact directly with glucose or ATP, but they are part of the active site of GK along with other neighbouring residues [31] [18] [32] [33]. His416 is located in the α11' helix in the large domain (Figure 4A). The larger size of the side chain of the arginine residue in the p.His416Arg mutation would be projected into surrounding α helices and could force a structural reorganization of that region of the protein (Figure 4B). Gly223 is localized in the ß9 strand, in the large domain (Figure 4A and 4C). The change of a small residue, glycine, to serine could also produce steric impediments with surrounding residues Thr206 and Met210 in the α5 helix, which are inside the active site and were previously found to be important for GK activity [19] [34] [35] [22]. In contrast to Asp205, His416 and Gly223, Ile130 is located in a peripheral position far from the catalytic site, in the α3 helix within the small domain (Figure 4A). This residue is projected into the hydrophobic core of the small domain. Mutation p.Ile130Thr introduces a polar residue in this hydrophobic region, which might alter the stability of the core and the structure and dynamics of the domain (Figure 4D). Accordingly, our biochemical analyses have shown that the decrease in Kcat is the main deleterious defect caused by mutation p.Ile130Thr on GK activity. Although affinity for glucose is not altered by this mutation, the affinity for the second substrate, ATP is increased. This latter effect was also observed for the MODY2 mutation p.Ser131Pro [36], suggesting a role of this region of the protein in the regulation of the affinity for this substrate.

The activity index obtained from the kinetic analyses of these mutants might be sufficient to explain the pathogenic effect of mutations p.Asp205His, p.His416Arg, p.Gly223Ser and p.Ile130Thr, since there is a direct relationship between this parameter and the glucose-stimulated insulin secretion threshold in a heterozygous mutant [19] [34]. However, this rule does not apply for mutation p.Ala449Thr since the corresponding activity index does not differ significantly from that of the wild type enzyme. This mutation did not confer thermal instability, suggesting that it does not affect the stability of the protein. In addition, possible defects in the regulation of this mutant by GKRP in the liver are unlikely since this mutation has no effect on the interaction of GK with GKRP. In contrast, the main kinetic characteristics of glucokinase, which play a key role in the function of this enzyme as a glucose sensor, *ie* low affinity for glucose and cooperativity, are lost in this mutant protein.

Ala449 is located in the α13 helix which is the C-terminal region of glucokinase (Figure 4A). The α13 helix forms part of the allosteric activator site and plays a key role in the conformational change between the active and inactive forms of glucokinase [18] [37] [38]. In contrast to other hyperglycaemia-associated GK mutations in α13 helix, mutation p.Ala449Thr did not appear to affect global GK activity. The increase in the Km value for ATP may not be relevant since it still remains below the intracellular ATP concentration [19]. The reduced Kcat is compensated by an increased glucose affinity and loss of cooperativity for this substrate, resulting in a catalytic efficiency (Kcat/S_0.5_^h^) similar to that of the wild-type (2.44±0.39 vs 2.29±0.23). In the structural model of GK, the side chain of alanine or threonine at position 449 is projected to the solvent in the super-open inactive conformation (Figure 4F). In contrast, these residues are facing the β sheet of the small domain in the closed conformation (Figure 4E). The larger and polar side chain of threonine at position 449 may alter the equilibrium between the conformational states of glucokinase. The high affinity for glucose exhibited by GST-GK(p.Ala449Thr) suggests that the closed conformation is stabilized in this mutant. This hypothesis is supported by an increase in affinity for GK inhibitor MH which binds to the catalytic site in the closed conformation of the enzyme [27]. Palmitoyl-CoA and GKRP appear to bind to distinct allosteric sites of the enzyme that could overlap in the hinge region and would act by interfering with the closure of the catalytic cleft [39] [29] [27]. Our results indicate that mutation p.Ala449Thr does not alter the interaction of GK with GKRP. However, the sensitivity to palmitoyl-CoA was increased in mutant proteins GST-GK(p.Gly223Ser) and GST-GK(p.Ala449Thr), suggesting that these mutations have some effect on the putative binding site for acyl-CoA esters in the large domain of the enzyme [29]. It has been proposed that GK activators avoid the super-open form and stabilize the open one to increase affinity for glucose and Vmax, respectively [18]. The compound LY2121260 binds to the allosteric activator site of the enzyme and activates the enzyme in a similar fashion [38]. The fact that mutation p.Ala449Thr decreases Kcat and S_0.5_ to a similar extent suggests non-productive substrate binding or blockage in substrate release, which could be due to an impairment of this mutant protein to return to its open form in order to release G6P and ADP [18]. Consistently, our results show that the compound LY2121260 had no further effect on GST-GK(p.Ala449Thr) affinity for glucose and slightly relieved the defect on catalytic constant.

In summary, the results of the biochemical analysis of these different MODY2-causing mutations illustrate the importance of the fine tuning of GK and its role in glucose sensing, and provide further insights into the role of the catalytic and allosteric activator sites in the regulation of the activity of this enzyme.
